# Resocialization mitigates depressive behaviors induced by social isolation stress in mice: Attenuation of hippocampal neuroinflammation and nitrite level

**DOI:** 10.1002/brb3.3604

**Published:** 2024-06-19

**Authors:** Hossein Amini‐Khoei, Hossein Tahmasebi‐Dehkordi, Elham Bijad

**Affiliations:** ^1^ Medical Plants Research Center, Basic Health Sciences Institute Shahrekord University of Medical Sciences Shahrekord Iran

**Keywords:** depression, neuroinflammation, nitric oxide, resocialization, social isolation

## Abstract

**Background and aim:**

Social isolation stress (SIS) is a stressor known to trigger depressive behaviors. Psychiatric disorders are associated with neurobiological changes, such as neuroinflammation and an increase in nitric oxide (NO) signaling. Despite the well‐established detrimental effects of SIS and the involvement of neuroinflammation and NO in depression, potential management strategies, especially resocialization, remain insufficiently explored. Our aim was to elucidate the effects of resocialization on depressive behaviors in socially isolated mice, with a focus on the possible involvement of neuroinflammation and nitrite in the hippocampus (HIP).

**Methods:**

We utilized 24 Naval Medical Research Institute male mice, maintained under both social and isolation conditions (SC and IC). After the isolation period, the mice were divided into two groups of eight, including the SIS group and a resocialized group. The SC group was kept without exposure to isolation stress. We conducted the open‐field test, forced swimming test, and splash test to evaluate depressive behaviors. Additionally, nitrite levels, as well as the gene expression of interleukin (IL)‐1β, tumor necrosis factor (TNF), and toll‐like receptor 4 (TLR4) in the HIP, were measured.

**Results:**

The study found that resocialization significantly reduces depressive behaviors in SIS mice. The results suggest that the antidepressive effects of resocialization may be partially due to the modulation of the neuroinflammatory response and nitrite levels in the HIP. This is supported by the observed decrease in hippocampal gene expression of IL‐1β, TLR4, and TNF, along with a reduction in nitrite levels following resocialization.

**Conclusion:**

These insights could pave the way for new management strategies for depression, emphasizing the potential benefits of social interactions.

## Graphical abstract

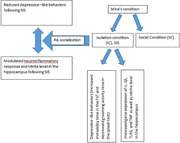

## 1 INTRODUCTION

Social isolation stress (SIS) is a significant stressor that can lead to the development of depressive behaviors (Haj‐Mirzaian et al., 2019; Xiong et al., 2023). In mice, SIS has been shown to induce a variety of behavioral changes, including increased anxiety and depressive behaviors (Ma et al., 2011). These behavioral changes are often accompanied by neurobiological alterations, such as increased in neuroinflammation and nitrite level in the brain (Haj‐Mirzaian et al., 2016; Joca et al., 2019; Mazrooei et al., 2023; Zheng et al., 2021).

Neuroinflammation, characterized by the activation of microglia and the production of pro‐inflammatory cytokines, has been implicated in the pathophysiology of depression (Wang et al., 2017). Among pro‐inflammatory cytokines, interleukin (IL)‐1β and tumor necrosis factor (TNF) play a significant role in the pathogenesis of stress‐induced depression (Arabi et al., 2021; Liu et al., 2015). TNF not only intensifies cell death and hinders neurogenesis (Manosso et al., 2013), but its levels are also found to be elevated in individuals diagnosed with psychiatric disorders (Bauer & Teixeira, 2019; Réus et al., 2015). Interestingly, blocking TNF has demonstrated antidepressant effects (Simen et al., 2006). Furthermore, TNF is involved in the induction of pro‐IL‐1β, which can eventually be converted to the active form of IL‐1β (Zhang et al., 2015). IL‐1β is known to suppress hippocampal neurogenesis and activate the hypothalamic‐pituitary‐adrenal (HPA) axis following stress, leading to the production of inflammatory mediators and contributing to the development of depressive behaviors (Cernackova et al., 2020). As a fundamental component of the innate immune system, toll‐like receptor 4 (TLR4), a variant of transmembrane protein, plays a crucial role in pattern recognition (Armant & Fenton, 2002). It is associated with the regulation of TNF through its signaling pathway (Chen et al., 2017). Furthermore, TLR4 has the ability to identify internal damage‐associated molecular patterns (DAMPs) (Pascual et al., 2021). This identification process instigates the production of pro‐inflammatory cytokines, including TNF, which are integral to the downstream signaling molecules of TLR4 (Liu et al., 2014). Indeed, neuroinflammation associated with DAMPs has been observed in the hippocampus (HIP), a brain region that plays a crucial role in the pathophysiology of depression (Klegeris, 2021; Serna‐Rodríguez et al., 2022; Troubat et al., 2021). Regarding pivotal role of HIP in the pathophysiology of depression, it has been determined that structural and functional changes in the HIP is involved in the pathophysiology of mood disorders like depression (Anjomshoa et al., 2020; Arabi et al., 2021).

Nitric oxide (NO), an endogenous activator of guanylyl cyclase, is derived from L‐arginine through the enzymatic action of NO synthase (NOS) (Knowles et al., 1989). It is involved in several physiological processes in the brain, including synaptic plasticity, neurogenesis, and neuronal survival (Amiri, Haj‐Mirzaian, et al., 2016; Contestabile & Ciani, 2004). Certain biological functions of NO are mediated by the soluble enzyme known as guanylyl cyclase, which subsequently leads to an increase in the levels of the secondary messenger, cyclic guanosine monophosphate(Krumenacker et al., 2004). It is currently understood that the NO and cGMP systems form the primary mechanisms for the effects of NO within the brain (Esplugues, 2002). NOS inhibitors, which have shown antidepressive effects, function by decreasing the levels of NO (Wegener & Volke, 2010). Furthermore, it has been demonstrated that inhibitors of NOS elevated extracellular serotonin and dopamine levels, key neurotransmitters associated with depression, in the HIP and subsequently mitigated depression, whereas l‐arginine, a precursor of endogenous NO, produces an opposing effect, indicating a potential negative regulatory role of NO on depression (Dhir & Kulkarni, 2011). Considering the aforementioned information and a substantial body of evidence, it is firmly established that NO plays a crucial role in the pathobiology of depressive disorders (Baranyi et al., 2015; Heydarpour et al., 2013; Kudlow et al., 2016).

Although the detrimental effects of SIS and the roles of neuroinflammation and NO in depression have been well documented (Mumtaz et al., 2018), less is known about the potential therapeutic strategies to mitigate these effects. Resocialization, or the reintroduction of social interactions, could be one such strategy (Li et al., 2021). However, the effects of resocialization on depressive behaviors, neuroinflammation, and NO signaling in socially isolated mice remain largely unexplored.

The present study explores the hypothesis that resocialization can mitigate the depressive behaviors induced by SIS in mice. The study further investigates the potential mechanisms underlying this effect, with a particular focus on the role of neuroinflammation and NO. The findings of this research could provide valuable insights into the neurobiological mechanisms underlying SIS‐induced depressive behaviors and offer potential therapeutic strategies for the treatment of depression emphasizing the potential benefits of social interactions.

## 2 MATERIALS AND METHODS

### 2.1 Ethics

The study's experimental procedures were carried out with rigorous adherence to the ethical standards detailed in the Guide for the Care and Use of Laboratory Animals (8th edition, National Academies Press), as recommended by the National Institutes of Health. The Shahrekord University of Medical Sciences’ Ethics Committee granted approval for the study protocol (Ethics Code: IR.SKUMS.REC.1398.099). Throughout the experimental procedures, careful measures were taken to minimize animal use as well as any potential distress to the animals, ensuring their welfare.

### 2.2 Study design

In this study, a total of 24 Naval Medical Research Institute male mice, obtained from the Pasteur Institute in Tehran, Iran, were used. Animals were kept under regular laboratory environment, including temperature: 22  ±  2°C, humidity: 50 % ±  10%, 12‐h light–dark cycle (with lights turned on at 8:00 a.m.), and ad‐libitum access to food and water. These mice, weighing 10–12 g and aged 21–25 days, were housed for three weeks under two distinct conditions: the social condition (SC) and the isolation condition (IC). For the SC, mice were grouped together in plexiglass box measuring 25 cm × 25 cm × 15 cm. This condition allowed for social interaction among the mice. The IC involved housing the remaining 16 mice individually. Each mouse was kept separately in plexiglass box measuring 24 cm × 17 cm × 12 cm, resulting in SIS. To minimize interaction and maintain isolation, the cages housing these animals were tidied up on a weekly basis by the same experimenter. All experiments were carried out between 10:00 a.m. and 02:00 p.m.

After three weeks, the mice in the IC were further divided into two sets of eight including IC mice without resocialization (kept individually in cages for 2 weeks further) and IC mice, which underwent resocialization. For resocialization, IC mice were regrouped in a plexiglass box for 2 weeks. The duration and approach for social isolation and resocialization were selected based on prior research (Amiri, Amini‐Khoei, et al., 2015; Drummond & Kim, 2023; Mazrooei et al., 2023). The mice that were kept under SC were considered the control group. In order to minimize potential biases in the experimental procedures and data interpretation, it is noteworthy that the experimenter responsible for conducting the behavioral experiments and subsequent molecular examinations was kept unaware of the group assignments. Behavioral experiments were conducted over three consecutive days following the SIS and resocialization period. We conducted all of our behavior experiments with the same animals in the following sequence (1) open‐field test (OFT), then (2) splash test, followed by the (3) forced swim test (FST). To maintain consistency and account for potential circadian fluctuations, all experiments were conducted between 9 a.m. and 1 p.m., aligning with the subjects’ typical active period. In order to prevent additional stress to the animals, the environmental and maintenance conditions were the same for all groups, and all behavioral tests were performed by one experimenter. At the end of study, the mice were euthanized under profound anesthesia using diethyl ether, and their hippocampi were extracted for subsequent molecular examinations (Khaledi et al., 2023).

### 2.3 Behavioral experiments

#### 2.3.1 Open‐field test

The OFT was used to clarify the locomotor function as well as anxiety‐like. Mice were placed individually on the central zone of the apparatus, and their behaviors were documented for 5 min. Distance moved and number of crossing by 4 feet from each square (horizontal activity) were assessed during 5 min (Amini‐Khoei et al., 2022). The OFT was done to assess the locomotor activity following different conditions (SC or IC). The OFT was performed immediately before the FST to consider ambulatory behavior as well as to confirm that adjustments that occur in motor activity did not affect the immobility time in the FST (Anjomshoa et al., 2020).

#### 2.3.2 Forced swimming test

The FST was utilized to evaluate depressive activities in mice. Each mouse was individually placed in a cylindrical container made of plexiglass, measuring 25 cm in height and 80 cm in diameter, filled with water maintained at a temperature of approximately 23–25°C. The depth of the water was such that the mice could not touch the bottom or escape. The test lasted for 6 min, with the first 2 min serving as an acclimation period and the remaining 4 min being the period of observation. The primary measure was the duration of immobility, where the mouse remained floating in the water, making only movements necessary to keep its head above water (Petit‐Demouliere et al., 2005).

#### 2.3.3 Splash test

The splash test was employed to assess self‐care and motivational behavior in mice, which are indicative of depressive behaviors. For this test, a 10% sucrose solution was sprayed onto the dorsal coat of each mouse using a spray bottle. Following the application of the sucrose solution, each mouse was then placed individually in a clean cage for a period of 5 min. During this time, the mouse's grooming behavior was observed and recorded. The primary measure was the total time spent grooming during the 5‐min observation period. A decrease in grooming time is considered indicative of a depressive state (Liang et al., 2008).

### 2.4 Nitrite assay

For the determination of nitrite concentrations, a colorimetric assay, grounded in the Griess reaction, was utilized. In this procedure, 100 μL of the homogenate samples of HIP were amalgamated with 100 μL of Griess reagent in each well. Following a 10‐min incubation period at ambient temperature, an automated plate reader was employed to gauge the absorbance at 540 nm. The nitrite concentration in each sample was then deduced using a standard curve of sodium nitrite supplied by Sigma. The nitrite level was determined using a standard curve of sodium nitrite (Sigma) and reported as micromole per mg protein (Wopara et al., 2021).

### 2.5 Real‐time polymerase chain reaction (RT‐PCR)

Total RNA was isolated from the HIP tissue using RNX‐plus. The quantity and quality of the isolated RNA were assessed using a NanoDrop spectrophotometer. The RNA was then reverse‐transcribed into cDNA using a PrimeScript RT reagent kit (Takara Bio, Inc.). Real‐time polymerase chain reaction was performed on the cDNA samples using a light cycler instrument (Roche Diagnostics) (Takara Bio) (Omidi‐Ardali et al., 2019). The results were analyzed using the 2^−ΔΔ^ *^Ct^* method to calculate the relative gene expression levels of TLR4, IL‐1β, and TNF in the HIP. The housekeeping gene B2M was used as a reference gene to normalize the gene expression levels. The genes and their primers are listed in Table 1.

**TABLE 1:** The genes and primer sequences used in polymerase chain reaction (PCR) amplification.

Primers	Forward sequence	Reverse sequence
B2M	TCATCGACACCTGAAATCTAGGA	TCATCGACACCTGAAATCTAGGA
IL‐1β	TCATCGACACCTGAAATCTAGGA	TCATCGACACCTGAAATCTAGGA
TNF	TCATCGACACCTGAAATCTAGGA	TCATCGACACCTGAAATCTAGGA
TLR4	TCATCGACACCTGAAATCTAGGA	TCATCGACACCTGAAATCTAGGA

Abbreviations: IL, interleukin; TNF, tumor necrosis factor; TLR4, toll‐like receptor 4.

### 2.6 Data analysis

The statistical analysis was performed using GraphPad Prism 8 software. Kolmogorov–Smirnov test was applied to evaluate the normal distribution of data, resulting in parametric data. Brown–Forsythe test was used for the evaluation of data homogeneity. Data were expressed as mean ± S.D and analyzed with one‐way variance analysis (ANOVA) followed by Tukey's post hoc test. Results were deemed statistically significant at *p* < .05. We set *α* error at 0.05 and power (1‐β) at 0.8, and the required total sample size per group was calculated as 8 in behavioral tests and 4 in molecular studies (Amiri et al., 2017; Haj‐Mirzaian et al., 2017).

## 3 RESULTS

### 3.1 Effect of SIS and resocialization on the immobility time in the FST

**FIGURE 1:**
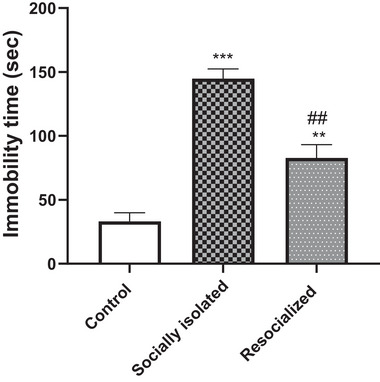
The impact of social isolation and resocialization on the immobility time in the forced swimming test (FST) was examined by calculating values based on a sample of eight mice per group, and the results were presented as the mean ± S.D. Statistical analysis involved the use of a one‐way variance analysis (ANOVA), followed by Tukey's post test. Significance levels were denoted as **p < .01 and ***p < .001 in comparison to the control group and ##*p* < .01 in comparison to the socially isolated group.

Figure 1 illustrates that the socially isolated group exhibited a significantly increased IN duration of immobility in the FST when compared to the control group (*p* < .001). Furthermore, resocialization led to a significant reduction in the duration of immobility when compared to the socially isolated group (*p* < .01). However, immobility time was significantly higher in the resocialized group than control group (*p* < .01).

### 3.2 Effect of SIS and resocialization on horizontal activity in the OFT

**FIGURE 2:**
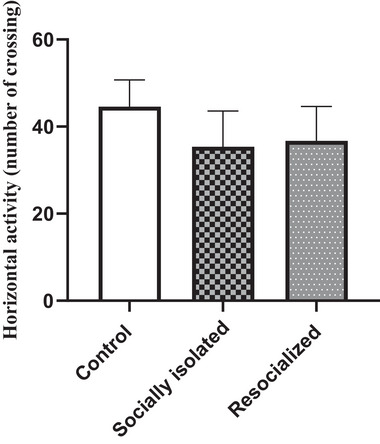
The impact of social isolation and resocialization on the horizontal activity in the OFT was examined by calculating values based on a sample of eight mice per group, and the results were presented as the mean ± S.D. Statistical analysis involved the use of a one‐way variance analysis (ANOVA), followed by Tukey's post test.

As depicted in Figure 2, the horizontal activity in the OFT did not undergo a significant change in the socially isolated group compared to the control group. Additionally, resocialization did not induce a significant alteration in horizontal activity compared to the socially isolated group.

### 3.3 Effect of SI and resocialization on the immobility time in the splash test

**FIGURE 3:**
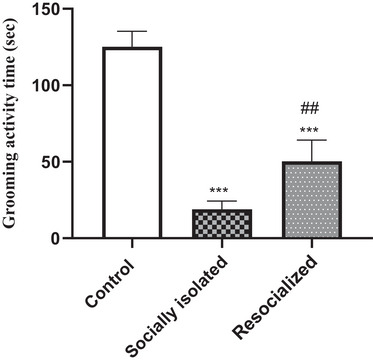
The impact of social isolation and resocialization on the grooming activity time in the splash test was examined by calculating values based on a sample of eight mice per group, and the results were presented as the mean ± S.D. Statistical analysis involved the use of a one‐way variance analysis (ANOVA), followed by Tukey's post test. Significance levels were denoted as ***p < .001 in comparison to the control group and ##*p* < 0.01 in comparison to the socially isolated group.

As illustrated in Figure 3, the grooming activity time during the splash test was significantly lower in the socially isolated group compared to the control group (*p* < .001). Furthermore, resocialization resulted in a significant increase in grooming activity time compared to the socially isolated group (*p* < .01). However, grooming activity time was significantly lower in the re‐socialized group than control group (*p* < .001).

### 3.4 Effect of SIS and resocialization on nitrite levels in the HIP

**FIGURE 4:**
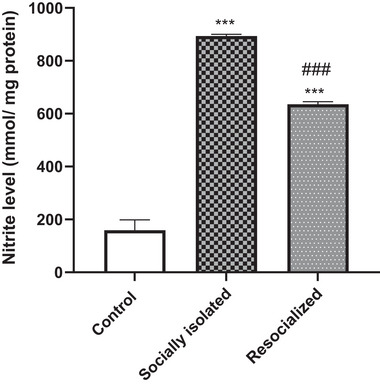
The impact of social isolation and resocialization on the nitrite levels in the hippocampus (HIP) was examined by calculating values based on a sample of four mice per group, and the results were presented as the mean ± S.D. Statistical analysis involved the use of a one‐way variance analysis (ANOVA), followed by Tukey's post test. Significance levels were denoted as ***p < .001 in comparison to the control group and ###*p* < 0.001 in comparison to the socially isolated group.

As depicted in Figure 4, the nitrite levels in the HIP were significantly higher in the socially isolated group than in the control group (*p* < .001). Additionally, resocialization resulted in a significant decrease in nitrite levels in the HIP compared to the socially isolated group (*p* < .001). However, the hippocampal nitrite level was significantly higher in the resocialized group than control group (*p* < .001).

### 3.5 The gene expression of IL‐1β, TLR4, and TNF in the HIP following SIS and resocialization

**FIGURE 5:**
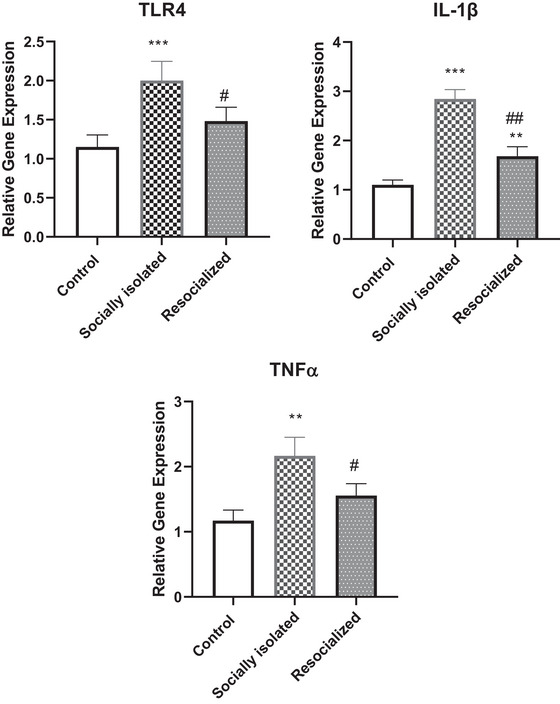
The impact of social isolation and resocialization on the gene expression of interleukin (IL)‐1β, toll‐like receptor 4 (TLR4), and tumor necrosis factor (TNF) in the hippocampus (HIP) was examined by calculating values based on a sample of four mice per group, and the results were presented as the mean ± S.D. Statistical analysis involved the use of a one‐way variance analysis (ANOVA), followed by Tukey's post test. Significance levels were denoted as **p < .01 and ***p < .001 and **p < .01 in comparison to the control group and ##*p* < .01 and #*p* < .05 in comparison to the socially isolated group.

Figure 5 illustrates the gene expression in the HIP for the socially isolated group and the effects of resocialization. The expressions of IL‐1β, TNF, and TLR4 were all significantly higher in the socially isolated group compared to the control group (*p* < .001, *p* < .01, and *p* < .001, respectively). Resocialization led to a significant decrease in the expression of these genes. Specifically, IL‐1β decreased significantly (*p* < .01), as did TNF and TLR4 (both *p* < .05). However, the gene expression of IL‐1β was significantly higher in the resocialized group than control group (*p* < .01).

## 4 DISCUSSION

The present study aimed to investigate the impact of resocialization on depressive behaviors induced by SIS in mice, with a focus on the potential involvement of neuroinflammation and nitrite levels in the HIP. The key findings of the study indicate that resocialization mitigates depressive behaviors induced by SIS. This was evidenced by a decrease in immobility time in the FST and an improvement in grooming activity time in the splash test. Additionally, the study discovered that SIS increased nitrite levels and upregulated gene expression of IL‐1β, TLR4, and TNF in the HIP, which were modified following resocialization.

The observed increase in immobility time in the FST in SIS mice is indicative of depressive behavior, corroborating previous research (Zanier‐Gomes et al., 2015). The subsequent decrease in immobility time following resocialization suggests that social interaction can counteract these effects, potentially offering a non‐pharmacological intervention for depression. This is in line with studies suggesting that environmental enrichment, which includes increased social interaction, can have antidepressant‐like effects (Brenes et al., 2008; Seffer et al., 2015). For instance, Seong et al. conducted a study using rat models of depression induced by chronic stress. Their findings demonstrated that environmental enrichment has a positive impact on hopelessness and anxiety levels as assessed through behavioral tests (Seong et al., 2017).

Evidences have determined that SIS is associated with anxiety‐like behaviors (Amiri, Amini‐Khoei, et al., 2015; Amiri, Haj‐Mirzaian, et al., 2015). It has been demonstrated that there are some potential overlaps between depressive‐ and anxiety‐like behaviors. Some symptoms of depression overlap with anxiety, and thus, tests focusing on these behaviors may display similar results in the effect of a treatments on anxiety and depression (Qureshi et al., 2021). In the other words, treatments may improve depression and anxiety symptoms because they are secondary to the SIS. However, in contrary, some studies have showed that some agents exert antidepressant effect but not anxiolytic effect (Haj‐Mirzaian et al., 2015). OFT is performed to evaluate the locomotor activity following different conditions (SC or SIS). The OFT was done immediately before the FST to consider ambulatory behavior as well as to confirm that adjustments that occur in motor activity did not affect the immobility time in the FST (Amiri, Amini‐Khoei, et al., 2016; Kaster et al., 2005; Lorigooini et al., 2021). OFT is also a valid tool for evaluation of anxiety‐like behaviors, in which time spent in central zone of the apparatus consider as an anxiety parameter (Lorigooini et al., 2020). One limitation of this study is that we did not evaluate time spent in central zone of the OFT. Thus, this parameter needs to be addressed in future studies to find potential overlap of depressive‐like behaviors with anxiety‐like behaviors as well as potential effects of resocialization on these.

The present study also found that SIS led to a decrease in grooming activity time during the splash test, another indicator of depressive behavior (Willner, 2005). The increase in grooming activity time following resocialization further supports the potential therapeutic benefits of social interaction. This is consistent with research showing that resocialization can modify the anxiety and depressive behavior induced by SIS (Kokare et al., 2010). For instance, Wilkin et al. by using male rats showed that the impact of intermittent physical stress (IPS) during mid‐adolescence on adult behavior was significantly influenced by housing conditions. Specifically, social housing, either continuous or after a period of isolation, was found to mitigate the negative long‐lasting effects of IPS, highlighting the stress‐reducing properties of social interactions (Wilkin & Menard, 2020).

In terms of neurobiological mechanisms, this study found that SIS led to an increase in nitrite levels and upregulation of IL‐1β, TLR4, and TNF gene expression in the HIP, suggesting a role for neuroinflammation and nitrite in mediating the effects of SIS. These findings align with a growing body of evidence implicating neuroinflammation and the nitrergic system in the pathophysiology of depression (Mazrooei et al., 2023; Troubat et al., 2021).

For instance, Kurosawa et al. (2016) showed that IL‐1β, particularly when coadministered with IL‐6, induces depression‐like behavior in mice, possibly through the activation of noradrenergic neurons in the locus coeruleus and elevated leptin levels. In a separate study, Hammad et al. (2021) found that chronic oxandrolone treatment led to similar behavior in rats, which was associated with a rise in proinflammatory cytokines (IL‐1β, IL‐6, and TNF) and a reduction in regulatory cytokine IL‐10 (Bugbee et al., 2023). Furthermore, in a study conducted by Alshammari et al., it was observed that SI led to a significant increase in the expression of TLR3, TLR4, and TLR7 genes in hippocampal tissue. Concurrently, the expression levels of IL‐6 and TNF were also upregulated. These findings suggest that SIS plays a substantial role in triggering neuronal inflammation (Alshammari et al., 2020). Elevated levels of TNF, IL‐6, and IL‐1β in depression may result from dysfunction in the brain's anti‐inflammatory mechanisms, including the HPA axis and noradrenergic innervation (Hannestad et al., 2011).

Furthermore, it has been observed that SI stress leads to the production of NO by neuronal NOS (nNOS). This in turn hinders neurogenesis in the HIP and disrupts brain signaling, thereby inducing depressive behaviors in animal models (Zhou et al., 2007). Aberrations in the nitrergic system, heightened oxidative state, and neurodegeneration have been associated with behavioral alterations observed in SI (Vrankova et al., 2021). In this regard, a study conducted by Gądek‐Michalska et al. (2019) revealed that SI stress could notably enhance the expression of the nNOS protein, potentially leading to an increase in NO synthesis within the HIP. In a separate study, Amiri et al. showed that the introduction of L‐NAME, a nonselective inhibitor of NOS, to socially isolated mice resulted in a decline in cortical nitrite levels and a reversal of the depressive behaviors induced by SIS stress (Amiri, Haj‐Mirzaian, et al., 2015). This is supported by the findings of this study, which showed that socially isolated mice had significantly higher nitrite levels in the HIP, a proxy for NO production (Salter et al., 1996), compared to control mice. This increase in NO could potentially lead to neuroinflammation, neuronal damage, and alterations in neurotransmission, which could manifest as depressive behaviors (Calabrese et al., 2007; Peng et al., 2012). Resocialization, on the other hand, appears to mitigate these effects. The present study found that resocialization led to a significant decrease in nitrite levels in the HIP of previously isolated mice, suggesting a reduction in NO production. However, the exact mechanisms through which resocialization reduces NO production and its subsequent effects on behavior are still not fully understood and warrant further investigation.

Studies have shown that the effects of SIS on the brain are not necessarily permanent and can be reversed. Environmental enrichment has been linked to numerous benefits, such as enhanced memory and reduced anxiety (Keloglan Musuroglu et al., 2022; Shahar‐Gold et al., 2013). Furthermore, social environments can promote neurogenesis and synaptic plasticity in the HIP (Biggio et al., 2019; Lu et al., 2003). In addition, resocialization has been found to normalize gene expression related to neuroplasticity in the amygdala (Lavenda‐Grosberg et al., 2022). These findings show that the structural and molecular alterations caused by SIS are modifiable through social interaction, underscoring the potential of resocialization as an effective strategy for counteracting the negative impacts of SI on the brain. The current study observed a decrease in IL‐1β, TLR4, and TNF gene expression, as well as nitrite levels in the HIP, following resocialization. These findings suggest that social interaction as resocialization may, at least partially, exert its antidepressive effects by modulating neuroinflammatory processes and nitrite in the HIP. In this regard, a study conducted by Gong and his team explored the effects of peripheral nerve damage during infancy on the emergence of anxiety and depressive symptoms in adolescent rats. The findings indicated that the introduction of an enriched environment could significantly mitigate these symptoms. This mitigation was achieved through the regulation of inflammation within the central nervous system. This was substantiated by the observed directional change in the elevated levels of IL‐1β and TNF and the reduced levels of IL‐10 (Gong et al., 2018).We did not assay IL‐6 to align with the past research mentioned above. However, many studies that aim to identify changes to pro‐inflammatory markers investigate the effects of IL‐6, and this remains a significant limitation of the current study.

Although the present study provides valuable insights, it is not without limitations. The use of only male mice limits the generalizability of the findings to female mice, who may respond differently to SIS and resocialization. Moreover, the study did not delve into the potential effects of varying durations or intensities of resocialization. Understanding the duration‐dependent and dose‐dependent impacts of reintegration into social environments could offer valuable insights for future research. Furthermore, it is imperative to acknowledge the potential lasting effects of SIS that may extend beyond the observed recovery period (Elizalde et al., 2008). For instance, it has been shown that isolation stress experienced during adolescence can significantly impact brain function, specifically affecting the HIP. This stress‐induced alteration may have enduring consequences, rendering the brain more susceptible to future insults (Hueston et al., 2017). However, the current research focused on a relatively short‐term resocialization intervention, leaving open the question of whether the positive outcomes are enduring or merely indicative of transient improvements. Addressing the longevity of these effects warrants consideration through longer‐term follow‐up studies that extend beyond the immediate post‐resocialization period. These investigations could explore the persistence or potential recurrence of depressive behaviors, neuroinflammatory markers, and nitrite levels over an extended timeframe. Furthermore, the exclusive use of male mice may limit generalizability. Future research should explore potential sex‐specific differences in effects of resocialization on depressive behaviors following SIS. Moreover, cortisol levels, integral to the stress response, were not investigated here. It is suggested that the relationship between levels of cortisol with depressive behavior and the effect of resocialization on cortisol levels should be investigated in future studies. Incorporating cortisol assays in future studies would enrich our understanding. Additionally, the focus on nitrite levels and specific gene expressions in the HIP leaves unexplored the roles of GABA, dopamine, and serotonin. Thus, role of these neurotransmitters in the effects of resocialization should be investigated in future assessments. Another limitation of our study is that we only evaluated IL‐1β, TLR4, and TNF at the gene level. Assessing IL‐1β, TLR4, and TNF at the protein level using western blotting, IHC, or ELISA is suggested for future studies.

## 5 CONCLUSIONS

In conclusion, this study provides compelling evidence that resocialization can alleviate depressive behaviors induced by social isolation in mice. The findings suggest that resocialization may exert its antidepressive effects, at least in part, by modulating neuroinflammatory processes and nitrite in the HIP. This is evidenced by the observed downregulation of IL‐1β, TLR4, and TNF gene expression, as well as decreased nitrite levels in the HIP following resocialization. These insights could pave the way for new therapeutic strategies for depression, emphasizing the potential benefits of social interaction.

## AUTHOR CONTRIBUTIONS

**Hossein Amini‐Khoei**: Conceptualization; investigation; funding acquisition; writing—original draft; methodology; validation; writing—review and editing; visualization; supervision; resources; project administration; data curation. **Hossein Tahmasebi‐Dehkordi**: Writing—original draft; writing—review and editing; methodology. **Elham Bijad**: Formal analysis; software; methodology; writing—original draft.

## CONFLICT OF INTEREST STATEMENT

The authors have no conflicts of interest to declare regarding the study described in this article and the preparation of the article.

## FUNDING INFORMATION

Shahrekord University of Medical Sciences, Shahrekord, Iran, Grant Number: 3533

### PEER REVIEW

The peer review history for this article is available at https://publons.com/publon/10.1002/brb3.3604.

## CONSENT TO PUBLISH

All authors reviewed and approved the manuscript.

## Data Availability

The datasets used and/or analyzed during the current study are available from the corresponding author upon reasonable request.
